# A Simple Modified Technique for Frameless Brain Lesion Biopsy

**DOI:** 10.7759/cureus.12002

**Published:** 2020-12-09

**Authors:** Sultan Al-Saiari, Ahmed A Farag, Khalid Al Orabi, Mohammad Abdoh, Hussein Kheshaifati

**Affiliations:** 1 Neurological Surgery, King Abdullah Medical City, Mecca, SAU

**Keywords:** burr hole, biopsy, frameless, navigation, technique

## Abstract

Published articles pertaining to possible ways to increase the accuracy of image-guided frameless surgery are abundant in the literature. Accurate target localization is dependent on many factors, of which noteworthy is the meticulous registration and constant fixation of instruments during the procedure. Frequent changing of instruments’ application or inadvertent destabilization of its fixation during surgery after registration might disrupt the preset navigation measurements, leading to inaccurate targeting. Technical wise, we managed to avoid the drawback of moving the aiming device repeatedly during the procedure, as we will discuss later.

This retrospective study aims to evaluate the feasibility and reliability of a simple frameless technique we used in navigation-guided brain biopsy and to show how it refines the accuracy of frameless biopsy procedures. All procedures were performed at our institution in the period from 2018 to 2019 and included 10 patients with different brain lesions. The mean operative time using our technique was noticeably short (18 minutes) and the standard deviation was 2.1. The used technique was easy, undemanding, and reliable in obtaining samples from brain tumors, guaranteeing more precision by applying an all-time fixed and stable navigation reference hardware.

## Introduction

Needle biopsy is a basic surgical procedure for definitive diagnosis of brain lesions especially for patients not fit for laborious craniotomies and those having contraindications to radical surgeries.

Image-guided techniques are now a hallmark in the field of neurosurgery. DICOM (Digital Imaging and Communications in Medicine) data that is uploaded to navigation software can be used to correlate instrument location in space to the preoperative image. A congruous spatial relation hence arises between surgeon, instrument, and the image software to finely target the lesion. Neurosurgeons are endeavoring to make surgery much delicate, accurate, and less damaging. Benefits include small incisions, short procedures, less injury to nearby structures, and rapid recovery [1].

Tissue diagnosis is vital for patients with brain tumors to determine future therapeutic plans and sometimes for prognostic implications. Diagnostic policies that depend only on imaging are not entirely definitive in diagnosis even with most advanced toolkits. Diagnostic accuracy of needle brain biopsy ranges from 83.6% to 100%, with morbidity and mortality varying from 0.7% to 16.1% and 0% to 3.9%, respectively [2]. It is advised to not use a frameless set to biopsy a lesion in the brain stem due to the unavailability of a device for secure fixation of the biopsy needle to reach deep lengthy trajectories [3].

## Technical report

Patients and methods

Patients’ Criteria

We identified 10 patients with different brain lesions who underwent a frameless navigation biopsy with the technical steps that will be described later. All surgeries were performed at our institution in the period from 2018 to 2019. Patient ages ranged from 16 to 70 years, with a mean of 55 years. Seven of them were males and three were females. Demographic data, radiological features, and histopathological diagnoses are summarized in Table 1.

**Table 1:** Demographic data, radiological features, operating time, and histopathological diagnoses.

Patient No.	Age/Gender	Location	Radiological Volumes of Lesion (mm)	Registration Time (min)	Number of Biopsies	Operating Time (min)	Pathological Diagnosis	Accurate Sampling of Lesion
1	70/F	Right anterior frontal	50	12	4	15	Glioblastoma	Yes
2	66/M	Left posterior frontal	45	9	4	18	Glioblastoma	Yes
3	55/M	Left posterior parietal	43	11	4	20	Grade III glioma	Yes
4	63/M	Right fronto-parietal	50	9	5	20	Glioblastoma	Yes
5	68/F	Right anterior frontal	55	10	6	16	Glioblastoma	Yes
6	59/F	Left thalamic	42	8	4	20	Glioblastoma	Yes
7	50/M	Left posterior frontal	52	11	5	17	Grade II glioma	Yes
8	16/M	Pineal region	53	10	4	20	Germinoma	Yes
9	58/M	Right fronto-temporal	57	9	4	15	Glioblastoma	Yes
10	45/M	Left temporo-parietal	60	11	6	19	Grade II glioma	Yes

All images were performed one day before surgery complying with the navigation protocol. Radiological volumes of lesions ranged from 20 mm to 50 mm with a mean of 30.7 mm on gadolinium-enhanced and FLAIR (fluid-attenuated inversion recovery) MRI.

We obtained multiple samples for each patient, which were all sent for frozen section pathology and were diagnostic.

A postoperative non-contrasted CT scan was performed for all patients.

In all cases, complete resection was not planned preoperatively due to different reasons such as multiple lesions, frail patients who cannot afford long surgery, lesions in eloquent areas that cannot be resected, and patients’ refusal of craniotomy. Initial pathological assessment was performed by a neuropathologist who examined different tissue samples. Multiple sampling was attempted until adequate tissue was verified. Tissue samples were preserved in 10% formaldehyde and sent for pathological identification. Histopathological diagnoses were based on the 2016 World Health Organization classification of central nervous system tumors [4].

Registration and trajectory identification

Registration was done as usual using an optical tracking system (StealthStation® S7®, Medtronic, Minneapolis, MN, USA). Intraoperative imaging registration and planning were done using a free hand-held multiplane pointer as a part of an optical tracking system (StealthStation S7). The head was fixed in the Mayfield® clamp (Integra LifeSciences, Princeton, NJ, USA), which was attached to the operating table to avoid movement. A dynamic reference arc was fastened to the operating table through a holder. When the surgeon became satisfied with the registration, the reference arc was removed and the draping of the patient ensued. It was feasible to allocate an entry point and a vector point to the trajectory of interest as it was done in registration. Alignment errors could be detected automatically and will show up on the computer screen. A new sterile reference arc was fixed as before. The Vertek® articulating arm (Medtronic) was pivoted to the dual starburst, which was secured to the head clamp on the same side of the lesion.

**Figure 1:**
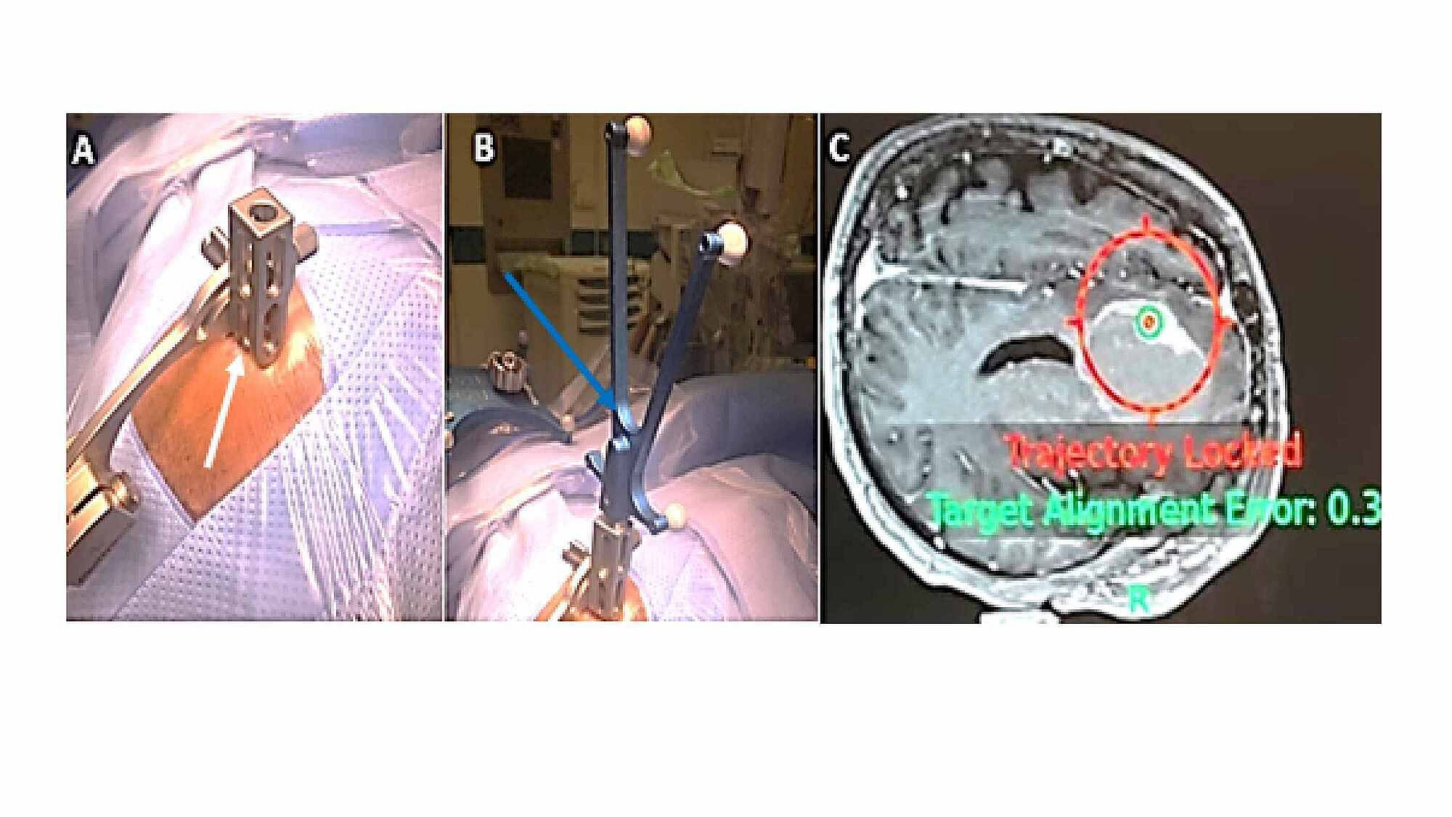
Mounting the aiming device (white arrow) to the skin entry point (A). Advancing the navigation probe (blue arrow) inside the skin entry point (B). Trajectory lock as represented by two congruent circles around the target (C).

Then, the aiming device was mounted to the skin entry point, ensuring to leave minimal space between it and the scalp point to avoid change in the predefined entry set registered resulting from thrusting the skin (Figure 1A). For trajectory alignment, the navigation Vertek probe was advanced through the aiming device smoothly to determine the perfect trajectory (Figure 1B). The probe was rotated until it was aligned with the trajectory of interest. The trajectory was then locked by pressing the footswitch, and the lock would show off as two congruent circles on the screen (Figure 1C). The pivot joints had also to be fastened tightly to secure the settled position.

Operative details

**Figure 2:**
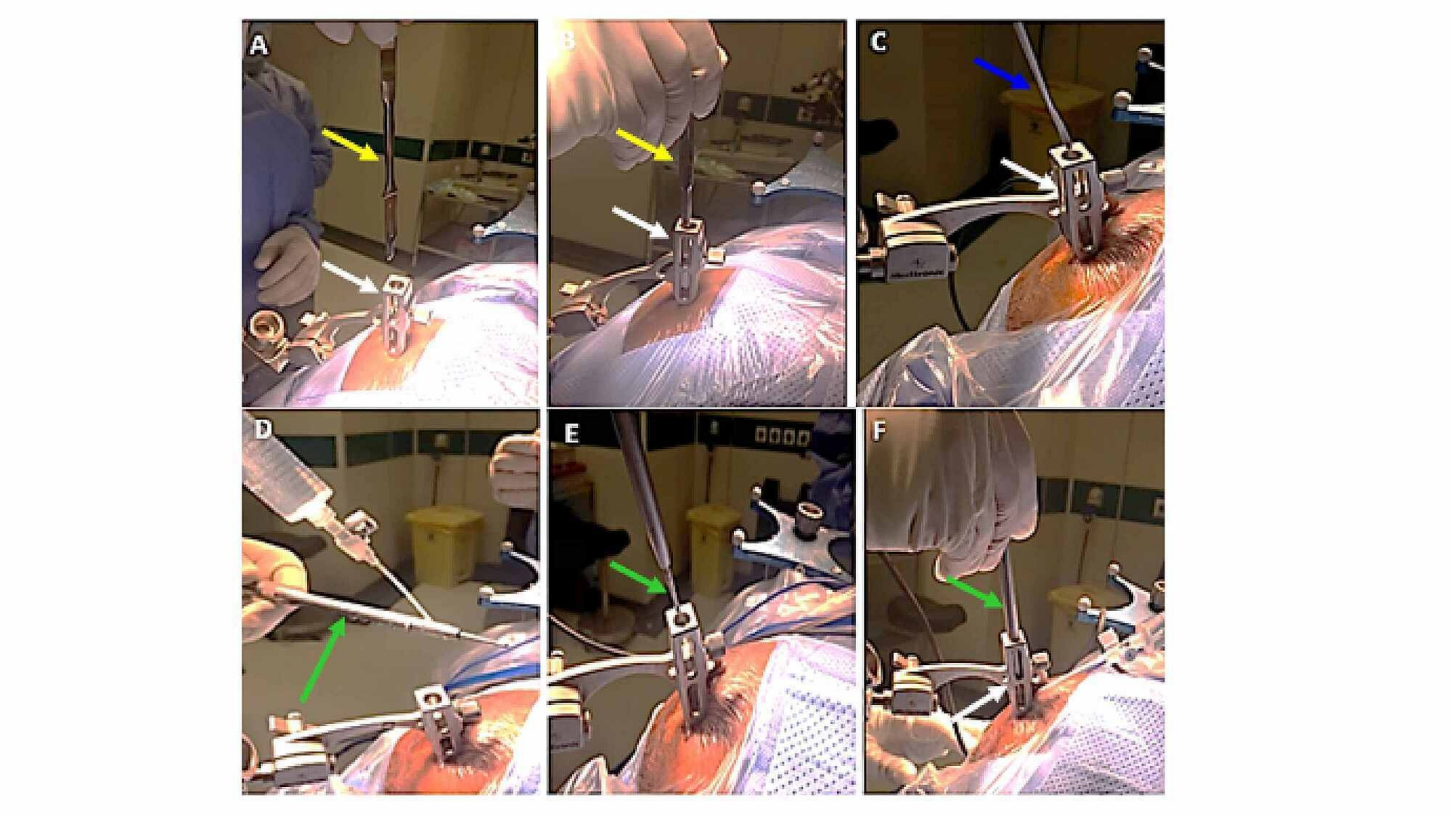
A scalpel knife (yellow arrow) size 7 was passed through the aiming device (white arrow) to incise the skin (A, B). A Penfield size 4 (blue arrow) was introduced to dissect the galea aponeurosis (C). A matched drill (14 cm in length and a 3-mm burr) (green arrow) is irrigated (D) and inserted through the aiming device (E, F).

**Figure 3:**
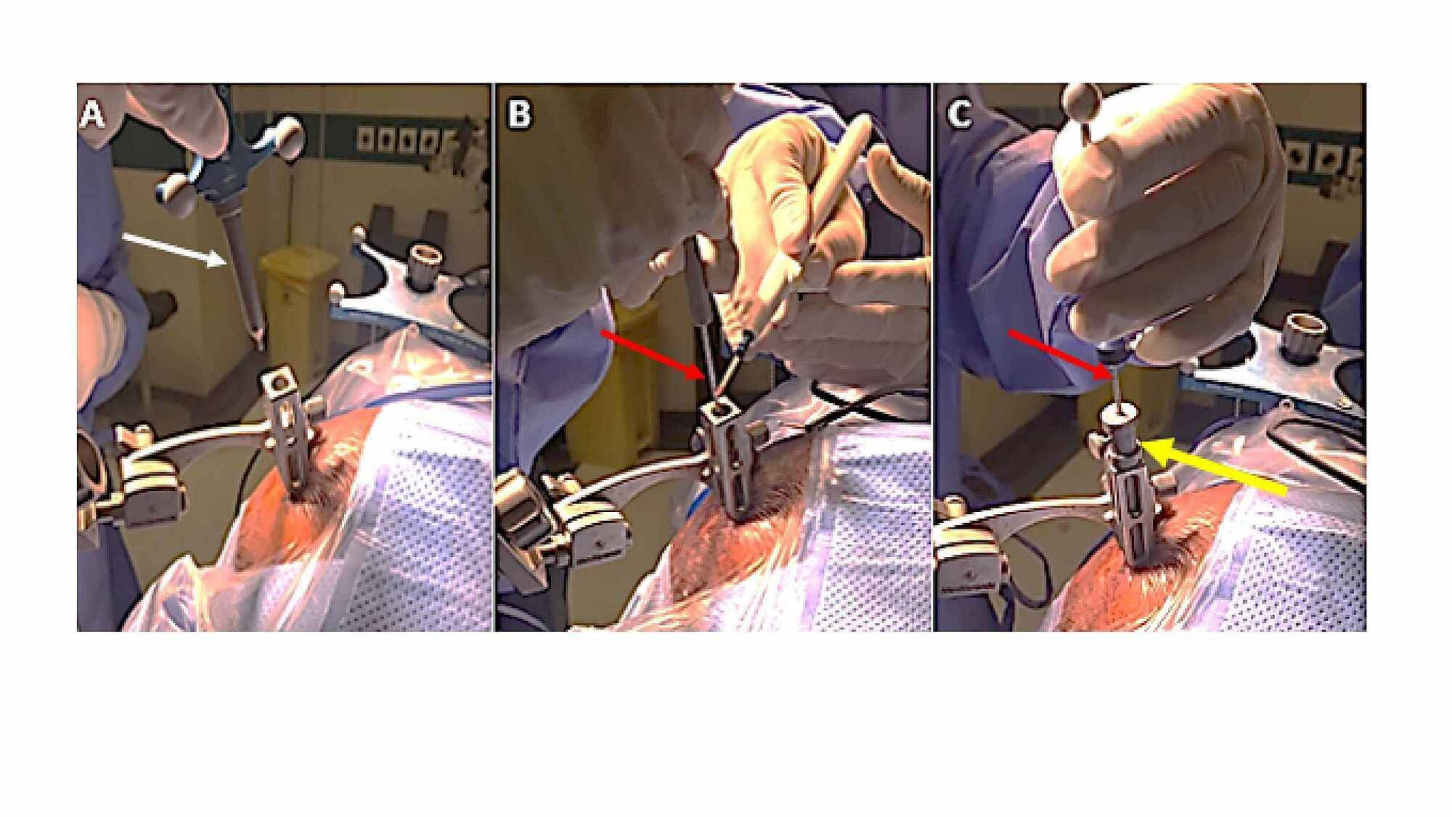
Reapplying the Vertek probe (white arrow) to ensure the proper alignment (A). Monopolar diathermy is applied to the needle biopsy (red arrow) to coagulate the dura (B). The biopsy needle (red arrow) is easily inserted through the reducing tube (yellow arrow) to reach the target.

A scalpel knife was passed through the aiming device to incise the skin (Figure 2A). A Penfield 4 was introduced to dissect the galea aponeurosis (Figures 2B, 2C). The matched drill shaft AA14 (Medtronic Midas Rex) with 14-cm length and a 3-mm burr was advanced to drill the skull entry point. Continuous irrigation was applied over the drill shaft (Figures 2D-2F). After drilling was done, we reapplied the Vertek probe to ensure proper alignment. We managed to coagulate the dura using monopolar diathermy applied to a closed mouth biopsy needle (Figure 3B). Diathermy was calibrated to 10 MA in all surgeries. All the previous instruments were advanced through the aiming device, which made the procedure neat, easy, and practical.

**Figure 4:**
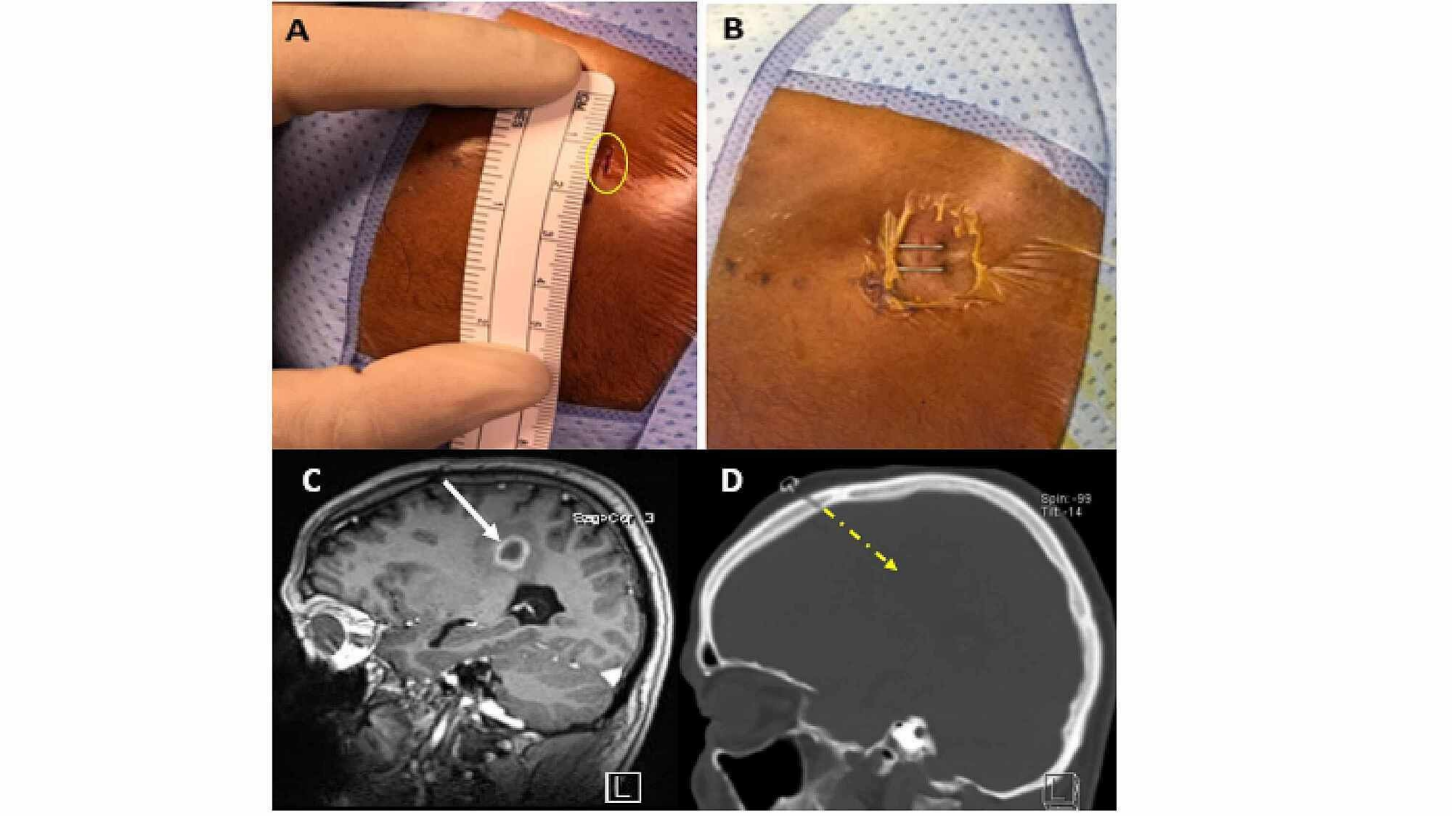
Incision size (A). Closure using two staples (B). Preoperative MRI (T1 with contrast) of a case with enhanced brain lesion in the left fronto-parietal region (white arrow) (C). A 3-mm burr hole as it appears in a postoperative CT head bone window, for the same case, with the trajectory direction (dotted arrow) (D).

A reducing tube with an inner diameter of about 2.2 mm was fitted and fixed to the aiming device channel. The biopsy needle was easily passed through the reducing tube (Figure 3C). The stop position of the biopsy needle was already calculated automatically, by navigational system, from the reducing tube to the target. Four to six biopsies were taken from different regions of the lesion in a clockwise direction (12, 3, 6, and 9) after the initial sample was confirmed by frozen section. Closure of the skin was performed by only one or two staples (Figures 4A, 4B).

Results

Preoperative system registration lasted 10 minutes as the average time. Operating time was measured by a staff nurse starting from skin incision to closure and ranged from 15 to 20 minutes, with a mean of 18 minutes and standard deviation of 2.1. If we added the time required for anesthesia to the procedure, it would range from 40 to 50 minutes. The number of biopsies ranged from four to six samples, with a median of four samples.

No major intraoperative complications were reported apart from breaching the dura, by the drill bit, in only two patients. Regarding postoperative complications, no postoperative problems were noticed among the patients. The median length of hospital stay was one day. No in-hospital or 30-day mortality was reported. The incision size was about 6 mm, the burr hole was almost 3 mm (Figure 4), and no skin depression was noticed. No wound-related problems were reported, and the healed wound could be hardly identified through the scalp in all patients.

## Discussion

Frameless navigation-guided biopsy is an advanced neurosurgical procedure that has a relatively better outcome compared to frame-based techniques [5]. Frameless methods acquired more efficacy in targeting brain lesions, especially the deeply located, for pathological diagnosis, with a degree of accuracy compared to frame-based stereotactic methods [6,7]. A common turmoil that usually faces surgeons while using frame-based stereotaxy is the application of the frame with the calculation of coordinates. It is a tedious process for both the surgeon and the patient, leading to extra time waste [8]. In opposition to rigid fixation in frame-based techniques, the frameless is sensitive to minimal hand tremors, drift, volume shift, and minimal movements of instruments. On the other hand, a common pitfall that encounters surgeons regarding frameless procedures is the structural shift resulting from debulking surgery that affects navigation measures relative to preoperative ones; however, it is uncommon to occur in biopsies performed through a burr hole.

The traditional technique usually used in frameless biopsy is summarized as following: after navigation is established and surgical vector alignment is secured adequately, the precision device is put aside, then the perforator is used to make a burr hole, then target alignment is readjusted using the Vertek probe, and the precision device is reapplied again. Those steps are repeated several times trying to reach the target in case the biopsy needle hits the inner table and the burr hole needs to be enlarged. The whole steps described are mind-numbing due to repeated detaching of the aiming device from scalp entry point, which eventually reduces the accuracy of navigation. The previously mentioned traditional steps consume more time and subsequently the surgeon becomes more distracted.

Concerning our technical point, navigating the target was done once from incision to dura coagulation and then drilling was performed through the precision channel without having to put it aside, use a perforator, or reapply the precision piece, which may again lead to changing its position that was already adjusted according to the predefined navigated trajectory. Shortcutting some steps in the traditional method described previously saves time and limits localization errors induced by frequent position changes of the precision piece while trying to undermine the inner table as the needle biopsy occasionally hits it. Ideal work in navigation surgery is determined by the ability of an instrument to reach a preselected target without deviation or injury to the normal brain. We used a simple technique that can minimize burr hole size using a 3-mm drill instead of using the cranial perforator. Burr hole that we used was about 3 mm in width, and we did not undermine any part of the inner table of the skull to widen it. We avoided hitting the inner table by aligning the burr hole entry point with the predefined trajectory (Figures 4C, 4D).

Patients are usually anxious about the wound and cosmetic complications, especially bold patients. Curtailing burr hole width to such a small size, as described, aided to alleviate psychological discomfort caused by wide skull holes. Furthermore, rapid wound healing allows the early treatment with radiotherapy when indicated. We managed to have a 3-mm burr hole as we described previously, the matched drill shaft can be forwarded through the precision device, and we did not use the perforator. Skin depressions observed in patients while combing hair are no longer observed in patients operated using this technique.

As we reviewed the literature, Parreño et al. used a technical step similar to that illustrated before [3]. The difference is that Parreño et al. used a custom-made tube through which they passed the 3-mm drill shaft, whereas we used a matched drill shaft to advance through the precision device to which it was adapted and easily coapted as discussed earlier.

## Conclusions

To summarize, this simple technique is a possible increment to improve the accuracy of initial operative navigation and limits the time consumed during navigational surgery. Anecdotal evidence from our experience suggests that it provides the surgeon with some finesse than the traditional biopsy technique we described previously.
